# Orienteering Problem with Functional Profits for multi-source dynamic path construction

**DOI:** 10.1371/journal.pone.0213777

**Published:** 2019-04-02

**Authors:** Ksenia D. Mukhina, Alexander A. Visheratin, Denis Nasonov

**Affiliations:** ITMO University, Saint Petersburg, Russia; National Taiwan University of Science and Technology, TAIWAN

## Abstract

Orienteering problem (OP) is a routing problem, where the aim is to generate a path through set of nodes, which would maximize total score and would not exceed the budget. In this paper, we present an extension of classic OP—Orienteering Problem with Functional Profits (OPFP), where the score of a specific point depends on its characteristics, position in the route, and other points in the route. For solving OPFP, we developed an open-source framework for solving orienteering problems, which utilizes four core components of OP in its modular architecture. Fully-written in Go programming language our framework can be extended for solving different types of tasks with different algorithms; this was demonstrated by implementation of two popular algorithms for OP solving—Ant Colony Optimization and Recursive Greedy Algorithm. Computational efficiency of the framework was shown through solving four well-known OP types: classic Orienteering Problem (OP), Orienteering Problem with Compulsory Vertices (OPCV), Orienteering Problem with Time Windows (OPTW), and Time Dependent Orienteering Problem (TDOP) along with OPFP. Experiments were conducted on a large multi-source dataset for Saint Petersburg, Russia, containing data from Instagram, TripAdvisor, Foursquare and official touristic website. Our framework is able to construct touristic paths for different OP types within few seconds using dataset with thousands of points of interest.

## Introduction

Orienteering problem (OP) is a class of routing problems, where the ultimate goal is to create a route through a given points of interest (PoIs), which would satisfy given conditions and constraints. Depending on domain PoIs can be touristic attraction places [1], crowdsourcing tasks [2] or safety places in case of emergency situations [3]. Diversity of possible applications led to a great variety of OP tasks from classic OP [4] and its generalization [5] to exotic ones dedicated to politician movements [6] or band tour planning [7]. But existing problems consider score of a certain place to be independent from other points in a route and from points of a search space, whereas in real life people when choosing place to visit usually take these aspects into account. For instance, after visiting several museums in a row user would likely prefer to have a break rather than to visit another museum, despite his or her personal interest in museums. Users also would choose next location in their route not from all locations in the city, but from those, which are more or less co-directional to a final location. To address this kind of scenarios we propose Orienteering Problem with Functional Profits (OPFP)—extension of classic Orienteering Problem, where node profit depends on a node position in the itinerary, other points in itinerary, and points in the dynamically changing search space.

Original orienteering problem is a special case of the traveling salesman problem [8] along with other specified routing problems—vehicle routing problem [9], bus routing problem [10], etc. It was proven to be NP-hard [11], and thus all OP modifications are also NP-hard. That is why scientists try to find the best solutions for this class of problems in terms of both solution quality and algorithm execution time. When it comes to solving orienteering tasks on a large scale, like construction of itineraries through locations in a big city [12], the need for an efficient solver becomes crucial, because search space can include tens or hundreds of thousands points. Modern algorithms for solving such problems consider amount of locations from dozens [13] up to one thousand [14], and time required to solve the problem for around 1000 locations in some cases is more than 30 minutes [15]. If we apply the same algorithms for larger datasets, execution time would go far beyond reasonable. This fact issues a great challenge for algorithms, which will be developed in this area. We propose solution for this problem by development of programming framework—FOPS (Framework for Orienteering Problem Solving)—that allows solving wide range of orienteering problems with high speed. Framework allows to develop implementations of its components in independent modular way so that created components could be used in other OP solutions with minimal changes.

To investigate computational efficiency of the developed framework for various problems in different conditions, we created a large multi-source dataset of locations for Saint Petersburg, Russia. It aggregates information from Instagram, TripAdvisor, Foursquare and official touristic guide. We performed thorough data processing in order to match locations from different datasets, remove incorrect locations and combine locations, which are related to the same place.

To sum up all described above, main contributions of the paper are the following:

1. Introduction and formalization of Orienteering Problem with Functional Profits.
2. Highly efficient open-source framework for solving the OP tasks—FOPS.
3. Large multi-source dataset of points of interest for Saint Petersburg, Russia.
4. Experimental results that demonstrate FOPS applicability on examples of five problems (including OPFP) of walking itineraries generation.

## Background

Problem of path construction with weighted nodes was introduced in work [4] as Orienteering Problem (OP). OP has various extensions [16] regarding different aspects of route construction. Team Orienteering Problem (TOP) involves constructing of multiple routes from start to terminal node [12]. In Orienteering Problem with Time Windows (OPTW) [17] availability of certain location is taken into consideration. Some places could be unavailable at certain time intervals. In Time Dependent Orienteering Problem (TDOP) [18] time required for visiting the node is considered for edge cost calculation and time for transition from one node to another depends on start time. A set of mandatory nodes, which are must-see, is included in Orienteering Problem with Compulsory Vertices (OPCV) [19]. Existing touristic paths can be successfully used for construction of new itineraries [20].

However, current researches of orienteering problems mostly consider complex constraints, whereas nodes scores have constant values. In Multi-Objective Orienteering Problem (MOOP) [21] nodes are combined to categories (e.g. landmarks, leisure, shopping) with different scores for each category. Thus, objective function was decomposed into several functions for each class, but category score function was similar to standard route score. Reward dependence on length of staying in some place was introduced in the Orienteering Problem with Variable Profits [22]. This idea was later expanded to objective function—score of a vertex was defined as time-dependent function in Periodic Traveling Politician Problem (PTPP) [6]. Another class of problems is Generalized Orienteering Problem with Resource Dependent Rewards (GOP-RDR), which was presented in [5]. In this type of problems reward collection at nodes and transition between nodes both consume some amount of a limited resource. In GOP-RDR score of each node is a vector of important factors such as money, fuel and so on. Total profit of concert tour was modeled using Attractive orienteering problem (AtOP) [7]. Benefit is defined as difference between profit from tickets sales and cost of concert and travel. It leads to more complex objective function where score of the current node depends on previous location. But still, for AtOP other factors of node profit remain constant. In [23], authors presented novel team orienteering problem with time windows and time-dependent scores. In this problem score of place also depends on the time of visit, for example, landscape view can be more attractive during sunrise or sunset and less attractive during night. However, in this formalisation authors considered only static periods of time i.e. morning, day, evening and did not include dynamic factors.

Another current direction of research is dedicated to novel formulations of existing problems. For instance, in [24] authors proposed generalization of classic OP, which gave rise to several new orienteering problems covering different aspects [5, 6]. Also, new formulations allow to achieve better results in terms of performance while preserving the solution quality. For example, in [25], authors proposed two integer linear programming formulations for classic OP. As a result both arc and node formalization demonstrated average computation time two times less than solving using original formalization. Vansteenwegen et al. [12] reviewed the most important variants of orienteering problems and their formulations; it was noted that several algorithms were optimized by utilizing information about OP formulations.

Despite active development in the field of orienteering problems, for the best of our knowledge there is no orienteering problem that fully covers dynamic profits of places. That is why we present formulation of Orienteering Problem with Functional Profits (OPFP) that tackles this issue. In OPFP score functions of particular node depends on three aspects: node’s characteristics, its position in the route, and other points included in the itinerary. Thus, solving OPFP allows to create complex orienteering scenarios, which are close to real-world ones.

### Algorithmic approaches for OP

The idea of automatic construction of the best route from one destination to another dragged a lot of attention from its beginning [26]. Orienteering problem was applied for itinerary design on different scales from parks [27] to cities [28]. This resulted in a diversity of methods for an optimal path construction. Path construction algorithm was proposed in [29], where authors presented several techniques to reduce execution time. Attraction Sorting mechanism was used to find an optimal sequence of points in a route. Results showed that improved algorithm requires 8 seconds for route generation for 250 attractions in comparison 1000 seconds for standard Dynamic Programming. Time-respected travel recommendation system for OPTW solving was described in [30]. Proposed approach consists of two phases: on the first stage route searching is performed to select the appropriate candidates; on the second stage heuristic algorithm is used to enhance the route. It was shown that for 300 PoIs, the itinerary can be constructed up to 2 seconds. Recursive greedy algorithm (RGA) presented in [31] was successfully used for Itinerary Mining Problem (variant of orienteering problem with node cost) [32]. Survey results showed that respondents prefer automatic itineraries to the real city tours.

Metaheuristic algorithms are widely used for solving different extensions of travel salesman problem [9, 33]. Scientists proved that metaheuristic algorithms are very efficient for solving OP and related problems [16]. It was shown in [23], that for orienteering problems with dynamic score the exact solution using integer linear programming formulations and specific software such as CPLEX require much more time for computations than heuristic algorithm. In survey [34] benchmarks for common algorithms were compared, and it was shown that ACO provides good results within appropriate time. Another solid comparison of different algorithms for solving orienteering related problems was presented in [15]. Authors investigated efficiency of several widespread algorithms. Experimental results demonstrate that ACO provides better results than other algorithms in terms of solution quality. In [35] fast ant colony system was presented for solving TD-OPTW, improved algorithm constructs solution in 0.4 s on the average for up to 100 nodes. It should be noted that due to the nature of the problem the node set is reduced to points which fit the time window.

In this paper we propose a programming framework, which can be a basis for creation of recommender system for touristic trips creation and can solve various types of problems with different algorithms depending on user needs. Due to the promising results and active use for OP family solving, we decided to implement the variation of RGA and ACO in our framework.

## Problem definition

Let us assume that *G* = < *V*, *E* > is a complete graph, where *V* = {*v*_1_…*v*_*N*_} is a set of locations with associated profit vector *s*_*i*_ ≥ 0, where *i* = 1, 2, …*N*, and *E* is a list of links between them with a cost vector *b*_*ij*_. The aim of solving OPFP is to find the most profitable and convenient way to reach terminate node from the start node whose total cost does not exceed a budget vector *B*. Formally, the resulting route is represented as an ordered sequence of vertices $C^{*}=\left\{v_{1},\ldots ,v_{u_{i}},\ldots ,v_{N}\right\}$ where *u*_*i*_ is position of vertex *v*_*i*_ in final route and *x*_*ij*_ represent the fact that route includes an edge *e*_*ij*_. Thus, OPFP can be formulated as an integer linear programming problem similar to other types of OPs [34]:

$$\text{max}\sum_{i=1}^{N-1}f\left(s_{i},C_{u_{i}-1}^{*}\right)\cdot \sum_{j=2}^{N}x_{ij},$$ (1)

subject to

$$\sum_{i=1}^{N-1}x_{iN}=\sum_{j=2}^{N}x_{1j}=1$$ (2)

$$\sum_{i=1}^{N-1}x_{ir}=\sum_{j=2}^{N}x_{rj}\leq 1,\forall r=2,\ldots ,N-1,$$ (3)

$$\sum_{i=1}^{N-1}\sum_{j=2}^{N}b_{ij}x_{ij}\leq B,$$ (4)

$$1\leq u_{i}\leq N,\forall i=1,2,\ldots N,$$ (5)

$$u_{i}-u_{j}+1\leq \left(N-1\right)\left(1-x_{ij}\right),\forall i,j=2,\ldots N,$$ (6)

$$x_{ij}=\{\begin{array}{cc} 1, & \text{if}\;\text{transition}\;\text{from}\;i\;\text{to}\;j\;\text{is}\;\text{in}\;\text{the}\;\text{route}\\ 0, & \text{otherwise;} \end{array},\forall i,j=1,\ldots ,N,$$ (7)

Objective function (1) is to maximize total profit of an itinerary. Total score of the route is sum of profits for each node. It is important to note that vertex profit depends not only on characteristics of a specific node *p*_*i*_, but on itinerary $C_{u_{i}-1}^{*}$ obtained by inserting previous node. Eq (2) ensures that path starts at node 1 and ends at node *N*. Similarly, constraint (3) ensures that resulting itinerary is connected and each node is visited only one time or not visited at all. Constraint (4) defines restrictions for the path and constraints (5) and (6) ensure that itinerary does not contain any subtours.

OPFP is NP-hard as an extension of classic OP. NP-hardness of OP was proven in [8].

## Framework for Orienteering Problems Solving

The majority of problems related to orienteering can be defined using three core components—set of PoIs, constraints and scoring function. The fourth core component—algorithm—utilizes other components in order to generate the best solution for a specific problem. We have developed a programming framework to make possible uniform and consistent development of algorithms for a wide range of orienteering problems—FOPS (Framework for Orienteering Problems Solving). Representations of four widespread OP types in terms of core components are available on GitHub (https://github.com/mukhinaks/fops). Main features of FOPS include:

1. Programming interfaces for core components, which include descriptions of corresponding functions.
2. Implementation of core components in an independent modular way for higher flexibility and reusability.
3. Single solving entry point—*Solver* class—that incorporates all core components.
4. Framework is developed with Go programming language (version 1.8.3), which is fast and provides great multithreading functionality.

**Fig 1:**
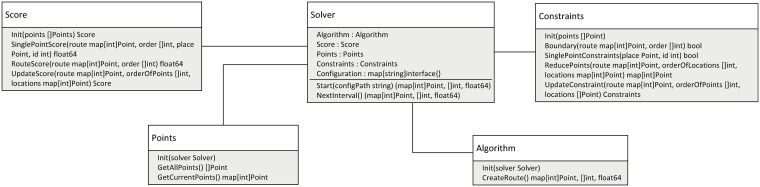
Architecture of the Framework for Orienteering Problems Solving.

In Fig 1 architecture of FOPS is presented. Main element of the framework is class *Solver*. It contains five entities, four of which are implementations of core components interfaces and the last entity is class *Configuration* that allows to set parameters of core components. The first method in *Solver* class is a *Start* function, which takes the path for configuration file as an input; this method is also responsible for initialization of core components instances by calling their *Init* methods. The second method is *NextInterval* which generates list of locations between two points and returns their final score as an output.

*Algorithm* interface includes two main methods. The first one—*Init*—is responsible for setting the initial state of the algorithm and saving the pointer to the *Solver* instance to be able to interact with other components during execution. The second method—*CreateRoute*—generates the route using the set of PoIs.

*Score* interface includes three main methods. *Init* sets the inner state of the scoring entity using a set of PoIs. Method *SinglePointScore* calculates a score for specific location based on its properties and relation to other points in the route. Method *RouteScore* is responsible for calculation of a total score of the generated route. Method *UpdateScore* return a copy of *Score* with updated parameters.

*Points* interface describes basic operations over a set of PoIs, based on which the solution is built. Method *Init* sets the inner state of the *Points* instance by reading data from a file or remote source, and saves the pointer to the *Solver* instance in order to achieve interaction with other components. Method *GetAllLocations* returns all PoIs from the set, whereas *GetCurrentLocations* filters locations with the help of constraints from the *Solver* instance.

*Constraints* interface includes five main methods. The first method—*Init*—sets the inner state of the constraints entity using a set of PoIs. Method *Boundary* is responsible for checking whether adding some PoI to the route violates any dynamic constraints of the problem, like time budget or money budget. Method *SinglePointConstraints* checks the matching of the PoI to the static constraints, e.g. check if the distance from the start point to the PoI being added is longer that the distance between start and end points. Method *ReducePoints* returns reduced set excluding location which do not satisfy problem constraints (for example current time windows) to decrease computation time. Method *UpdateConstraint* ensures parameters adjustment if necessary.

Source code of FOPS is available on GitHub (https://github.com/mukhinaks/fops). Implementations of all core components developed for experiments, are also included in the repository. Firstly, in combination with the dataset of PoIs, it makes the results of our research totally reproducible and easy to compare with any other solutions developed within the framework. And secondly, implementations included in the framework can help researchers, who would like to use FOPS, to get familiar with it and better understand the inner mechanics of the framework.

### Algorithms

#### Ant Colony Optimization

Ant Colony Optimization (ACO) is widely used for solving orienteering problems, because it provides high quality solutions in reasonable time and naturally correlates with the idea of searching the best route. The probability of choosing location *i* with previous location *j* is defined by the following equation:

$$p_{ji}=\frac{\tau_{ji}^{\alpha }\cdot f_{i}^{\beta }}{\sum_{k=1}^{N}\tau_{ji}^{\alpha }\cdot f_{i}^{\beta }},$$

where *α*, *β* are control parameters of the algorithm, $f_{i}=f\left(s_{i},C_{u_{j}}^{*}\right)$—node profit and *u*_*i*_ = *u*_*j*_ + 1;

$$\tau_{ji}=\left(1-\rho \right)\cdot \tau_{ji}+\sum_{ants}\Delta \tau_{ji},$$

*τ*_*ji*_ is the amount of pheromones deposited by single ant and *ρ* is pheromones evaporation coefficient. Δ*τ* is a route score normalized by number of ants.

$$\Delta \tau_{ji}=\{\begin{array}{cc} \frac{\sum_{\forall u\leq u_{j}+2}f\left(s_{k},C_{u}^{*}\right)}{N}, & \;\text{if}\;\text{the}\;\text{constructed}\;\text{path}\;\text{satisfies}\;\text{time}\;\text{conditions}\\ 0, & \text{otherwise;} \end{array}$$

where *u*_*j*_ + 2 = *u*_*N*_ (*N* is the terminal node), and *k* is index such that *u*_*k*_ ∈ {1, …*u*_*N*_}.

Ant repeats the process of a new place selection until the end location *N* is reached or the constructed path exceeds time limit. When all ants finish, the next iteration begins. Control influence parameters are usually varied between 1 and 5 [36], in this work *α* = 2 and *β* = 3, evaporation coefficient is set to *ρ* = 0.1 since it is a common parameter for solving problems of similar types [37]. The number of ants in a colony is dynamic and depends on cardinality of |*V*| = *N* and equals to *n* ⋅ *N* ants, where n∈R and *n* > 0. It was shown that for a large number of points in the dataset ACO performs better for the bigger number of ants starting from *n* = 0.25 [38]. Thus, the number of ants is defined as *n* = 1 per PoI and the number of iterations is 100. Due to the stochastic nature of ACO, the path with the best score from all iterations is taken.

#### Recursive Greedy Algorithm

The RGA was stated in [31] for searching optimal path in directed graph. It was used for itinerary construction in [32]. The main idea is find the best middle node and recursive call node search for both halves of route. The implementation used in FOPS slightly differs from original algorithm, see Algorithm 1. On each step, algorithm tries to insert a best node between all pairs of nodes and only nodes which maximize total route score will be added. It helps to manage situations where all highly-evaluated nodes are located in particular area. Candidate nodes are selected from a search space which is formed by *checkConstraint* method. For example, for OPTW search space consists of available locations only.

**Algorithm 1** Recursive greedy algorithm

1: **procedure** RGA(*route*, *B*)

2:  **if** *cost*(*route*) > *B* **then return** *route*

3:  **for** *u* ≔ 1, *u* ≤ *length*(*route*) − 1 **do**

4:   *V*_*u*_ = {*v* | *checkConstraint*(*v*, *route*), *v* ∈ *V*}

5:    **for** each *v* ∈ *V*_*u*_ **do**

6:    *testRoute* ← (*v*_1_, …, *v*_*u*_, *v*, *v*_*u*+1_…*v*_*N*_)

7:    **if** *cost*(*testRoute*) ≤ *B* **then**

8:     **if** *prize*(*testRoute*) ≥ *prize*(*route*) **then**

9:      *route* ← *testRoute*

  **return** RGA(*route*, *B*)

## Dataset formation

To solve OPFP on a city scale, we required large and high-quality dataset containing data from multiple sources. With spreading of the Internet all around the world, social media now play a major role in tourism area from marketing aspects to information search and decision making [39]. Foursquare and Instagram are widely used for urban studies and for studying tourists and city residents behavior in particular [40]. Combination of multiple sources, Flickr and Wikipedia, was used in TripBuilder [41]. However, Wikipedia provides information only about a number of relevant places and limited by historical and well-known locations. But during city development buildings could be destroyed or organization may move to another place, and these factors lead to potentially outdated itineraries. Touristic resources such as TripAdvisor, Yahoo Travel or Lonely Planet [32] are used for itinerary construction as a filter for larger spatial datasets, like Flickr and Instagram. However, this results in a significant reduction of resulting datasets, and some interesting and popular places are lost due to their absence in city guides. That is why when creating combined dataset from diverse data sources it is very important to augment data instead of truncating it.

**Fig 2:**
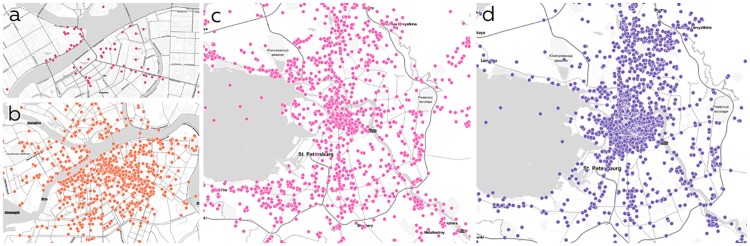
Coverage of Saint Petersburg by different sources. a—official city guide, b—TripAdvisor, c—Instagram, d—merged dataset.

Official city guide “Visit Petersburg” (http://www.visit-petersburg.ru), TripAdvisor, Foursquare and Instagram were chosen to ensure the collected data will cover all possible interesting places. Only publicly available data concerning venue information (coordinates, categories, address, open hours and title) was collected from official city guide, TripAdvisor, Foursquare, and Instagram. No personal data were collected or used during this study. Comparison of raw datasets coverage is presented on Fig 2. Notable advantage of social networks is their internationality and availability, i.e. datasets similar to the one used in our work could be created for every relatively large town in the world.

TripAdvisor contains basic information, such as address and open hours, about all places, which are popular among tourists. Tourists can leave a feedback about some place and evaluate it on scale from 0 to 5. For Saint Petersburg, TripAdvisor dataset contains 1736 ‘things-to-do’ with 10893 locations in total including restaurants and hotels.

Foursquare is based on the same idea that people can mark places where they have been and write a review. However, Foursquare started as a social network and encouraged users to check-in in different places. Group of superusers can change information about venues which results in a wide range of places. Foursquare provides basic information (address, open hours, tips, rating, etc.) for its venues. Dataset from Foursquare contains 5917 places.

Instagram proved itself as a fine source for restoring data about city [42]. Instagram is one of the largest and fast-growing social networks; by the end of 2017 Instagram had 800 million users with 500 million active every-day users from all over the world. Instagram locations are based on Facebook pages, which means that users are free to create their own places. This results in situation where some places have duplicate points for different languages. For this reason, places with Cyrillic and Latin names only were kept in dataset, but there is a small set of places with mistyped or synonymic names. Nonetheless, these places have a small number of visitors and low marks, so someone could duck this issue by choosing appropriate score function. Thus, the raw Instagram data contains 6436 locations.

**Fig 3:**
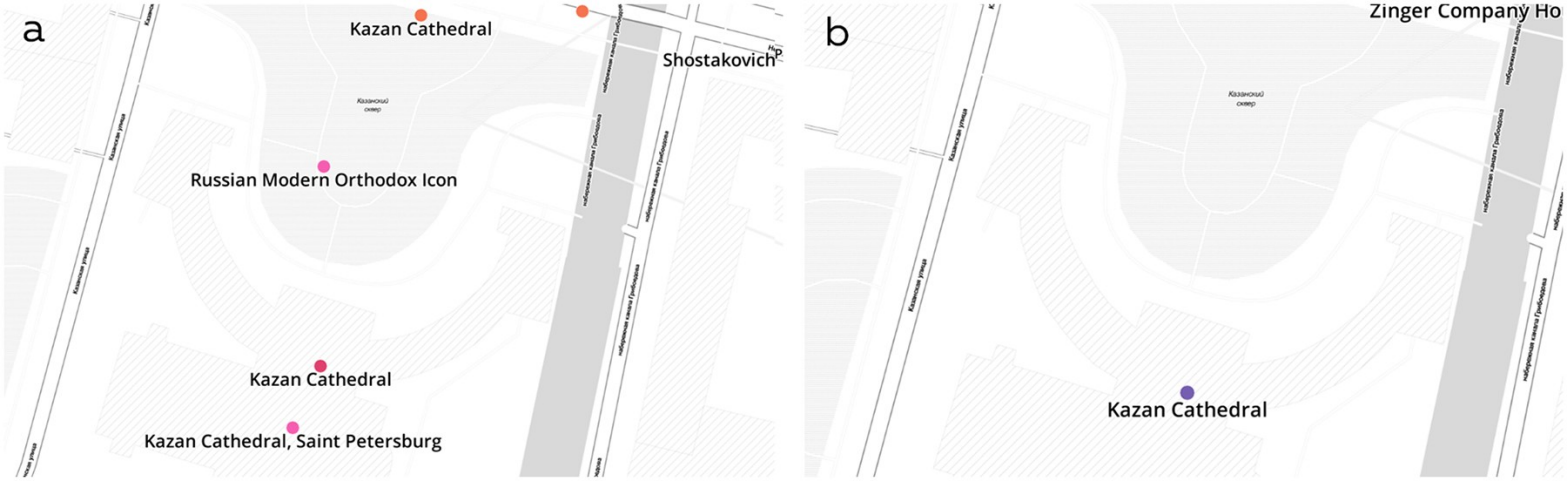
Data processing example. a—PoIs from raw datasets: blue color indicates locations ‘Visit Petersburg’, yellow—places from TripAdvisor, and pink indicates Instagram places; b—aggregated data.

The main problem of using datasets from different sources is to combine them correctly. Since the Internet resources set geographical coordinates by themselves, there is a typical situation when different data points correspond to the same place (Fig 3a). It is also possible that title of one place is a substring of another (e.g. the Alexander Theater and New Stage of the Alexander Theater are two different places). However, in this approach tourists have zero information about an area what they are going to visit and what is unique about the particular site. That is why it was decided to use places themselves as attraction points.

Thus, the final list of locations was created through several steps. First, for datasets from Foursquare, TripAdvisor and Official City Guide, it was assumed that they consist of unique elements only. For Instagram places, information about addresses, names, and coordinates was extracted from Google using Google Places API. Points with exact match of address, coordinates and name were considered as the same place (Fig 3b). Aggregated location name, address, coordinates and open hours were defined considering this priority: Official City Guide, TripAdvisor, Foursquare, and the most popular Instagram place.

To propose reasonable duration of visit in some area, it was decided to divide all places into three classes: Nature & Parks, Sights & Landmarks, and Museums & Libraries. Categories outside this list were excluded from analysis for experiments; however, the complete dataset was used for combining all data to obtain as much information as possible about every place. Each category has its own estimated time of visit [41]: 15 minutes for Sights & Landmarks, 1 hour for Nature & Parks, and 2 hours for Museums & Libraries.

Nevertheless, there are several aspects of the dataset, which must be noted. First, some locations still could be found with modified names and slightly different coordinates in every dataset; for example, the Winter Palace (also known as Winter Palace of Peter I) is a building where the Hermitage museum is located, and thus they will represent different places while having the same location. However, this kind of distinction has an advantage in case of architectural merit buildings where tourists potentially want to spend time on sightseeing. Second, proposition about uniqueness of places and correctness of information in Foursquare and TripAdvisor is not completely true. So, several TripAdvisor places has default coordinates at city center; these locations were excluded from the dataset. Some of Foursquare locations have wrong open hours intervals, this could be fixed by usage of reliable data source such as official site of location, official Facebook page or information from Google Places API. To summarize all described above, the complete dataset contains 5368 places and it is available online (https://doi.org/10.7910/DVN/KCAIXS).

## Experiments

### Score function

Due to the nature of the dataset, attractiveness of a certain point was defined as following score function:

$$f\left(s_{i},C_{u_{i}-1}^{*}\right)=\left(w_{i}^{inst}+w_{i}^{fsq}+w_{i}^{spb}+w_{i}^{trAdv}\right)+r_{i},$$

where $s_{i}=\left(s_{i}^{inst},s_{i}^{fsq},s_{i}^{spb},s_{i}^{trAdvRating},s_{i}^{trAdvReview}\right)$ is profit vector of node *i*;

$w_{i}^{inst}=\frac{s_{i}^{inst}}{\text{max}\;s^{inst}}\in \left[0;1\right]$, where $s_{i}^{inst}$ is the amount of Instagram visitors in point *i*, max *s*^*inst*^ is the maximum amount of visitors in selected area between points with indexes *u*_*i*_ − 1 and *u*_*i*_ + 1;

$w_{i}^{fsq}=\frac{1}{10}\cdot s_{i}^{fsq}$, where $s_{i}^{fsq}\in \left[0;10\right]$ is the Foursquare rating in point *i*;

$$w_{i}^{spb}=s_{i}^{spb}=\{\begin{array}{cc} 1, & \text{if}\;i\;\text{is}\;\text{listed}\;\text{in}\;\text{the}\;\text{official}\;\text{guide,}\\ 0, & \text{otherwise}; \end{array}$$

$w_{i}^{trAdv}=\frac{1}{5}\cdot s_{i}^{trAdvRating}\frac{s_{i}^{trAdvReview}}{\text{max}\;s^{trAdvReviews}}$, where $s_{i}^{trAdvRating}\in \left[0;5\right]$ is TripAdvisor rating, $s_{i}^{trAdvReview}$—number of reviews for location *i*, and max *s*^*trAdvReview*^ is maximum amount of reviews in the area between points with indexes *u*_*i*_ − 1 and *u*_*i*_ + 1;

$$r_{i}=\{\begin{array}{cc} \frac{\ell (u_{i}-1,u_{i}+1)}{\ell \left(u_{i}-1,u_{i}\right)+\ell \left(u_{i},u_{i}+1\right)},\\ 0, & \text{if}\;\ell \left(u_{i}-1,u_{i}+1\right)<\ell \left(u_{i},u_{i}+1\right)\;\text{or}\;\\ & \ell \left(u_{i}-1,u_{i}+1\right)<\ell \left(u_{i}-1,u_{i}\right) \end{array}$$

where *ℓ*(*u*_*i*_ − 1, *u*_*i*_ + 1) is distance between points with indexes *u*_*i*_ − 1 and *u*_*i*_ + 1, *u*_*i*_ + 1 indicates next point of the route (could be the last point *N* depending on the construction algorithm) and *j* such that *u*_*j*_ = *u*_*i*_ − 1 is the previous point from the route. In orienteering problems, appropriate path length is assured by satisfying time budget *B* (constraint (4)), but this is a superior limit. To achieve the shortest path in terms of budget, distance parameter was included into score function. For instance, in case of walking path generation, it is required to obtain convenient path, where all locations are close to each other and lead to the finish point.

In the meantime, it should be noted that $f(s_{i},C_{u_{i}-1}^{*})$ is varied in the interval (0; 5] since *r* always greater than 0 and at least one from the sum of $w_{i}^{inst},w_{i}^{fsq},w_{i}^{spb},w_{i}^{trAdv}$ is distinct from zero, otherwise the place won’t be on the list. Final route score is defined as total sum of scores $f(s_{i},C_{u_{i}-1}^{*})$ of all locations.

### Comparison of route construction algorithms

For studying of computation time, first, four locations were randomly selected from the dataset. Two locations were used as start and end points similar for all orienteering tasks, and other two were used as compulsory locations for OPCV. After that, there were generated 6 random samples from original dataset with various sizes: 10, 50, 100, 500, 1000 and 5000 places. Time limit was 10 hours and for TDOP and OPTW starting time was 10:00 a.m. Since the standard formulation of OP, OPCV, OPTW, and TDOP and their solving algorithms do not assume any computation for node score, number of Instagram visitors was taken as a single vertex score, this popularity score is widely used in these tasks [32, 43, 44]. Thus, in case of OP, OPCV, OPTW, and TDOP, the route score was computed as the sum of Instagram visitors from all places and for OPFP, the final score was computed as the sum of scores described in previous section. All experiments were launched 50 times on the workstation with Intel(R) Core i7-3930K @ 3.20GHz processor, 32 GB RAM, Windows 7 SP1. As it can be seen from Table 1, with increasing number of locations in a sample final score grows significantly. For the set of 100 vertices, ACO requires in average approximately a second for finding a best solution (Table 2), which goes with the best results in this area [35] for OPTW and TDOP problems. In [45] it was shown that for dataset of 400 nodes, it is required 2 seconds for solving OP. In addition, authors tested performance of well-known algorithms on a large dataset (7397 nodes); the best result was obtained for EA4OP algorithm—990.42 sec. Results obtained by ACO comply with these numbers. In some cases, RGA shows a bit worse results in terms of solution quality, due to the fact that algorithm can stuck in local maximum. However, in situations where computation time is crucial, e.g. for mobile applications, it can be successfully used.

**Table 1:** Average final route score for different OPs.

	10	50	100	500	1000	5000
Ant Colony Optimization						
OP	1038	74928	76546	213162	107519	774509
OPCV	830	16579	71489	61544	80377	518948
OPTW	1038	74949	76348	214212	84235	818200
TDOP	1035	74985	77785	213736	105130	778343
OPFP	6.31	7.97	9.19	18.5	19.77	25.52
Recursive Greedy Algorithm						
OP	1868	76063	73633	214593	117008	819030
OPCV	1990	76315	84140	205751	86288	705874
OPTW	1868	76063	73633	214593	107330	785090
TDOP	1868	77117	73461	214916	123248	825246
OPFP	7.8	4.45	5.76	19.5	16.77	22.62

**Table 2:** Average computation time in seconds.

	10	50	100	500	1000	5000
Ant Colony Optimization						
OP	0.01	0.08	0.19	4.93	13.14	243.25
OPCV	0.04	0.22	0.55	7.02	28.5	669.46
OPTW	0.01	0.09	0.22	4.98	9.78	280.01
TDOP	0.01	0.08	0.2	5.15	12.73	240.97
OPFP	0.01	0.05	0.14	3.59	11.96	220.85
Recursive Greedy Algorithm						
OP	0.001	0.004	0.014	0.04	0.08	0.35
OPCV	0.001	0.004	0.007	0.04	0.07	0.41
OPTW	0.001	0.003	0.011	0.04	0.08	0.42
TDOP	0.002	0.004	0.01	0.05	0.11	0.49
OPFP	0.001	0.003	0.01	0.10	0.11	0.75

### Routes examples

**Fig 4:**
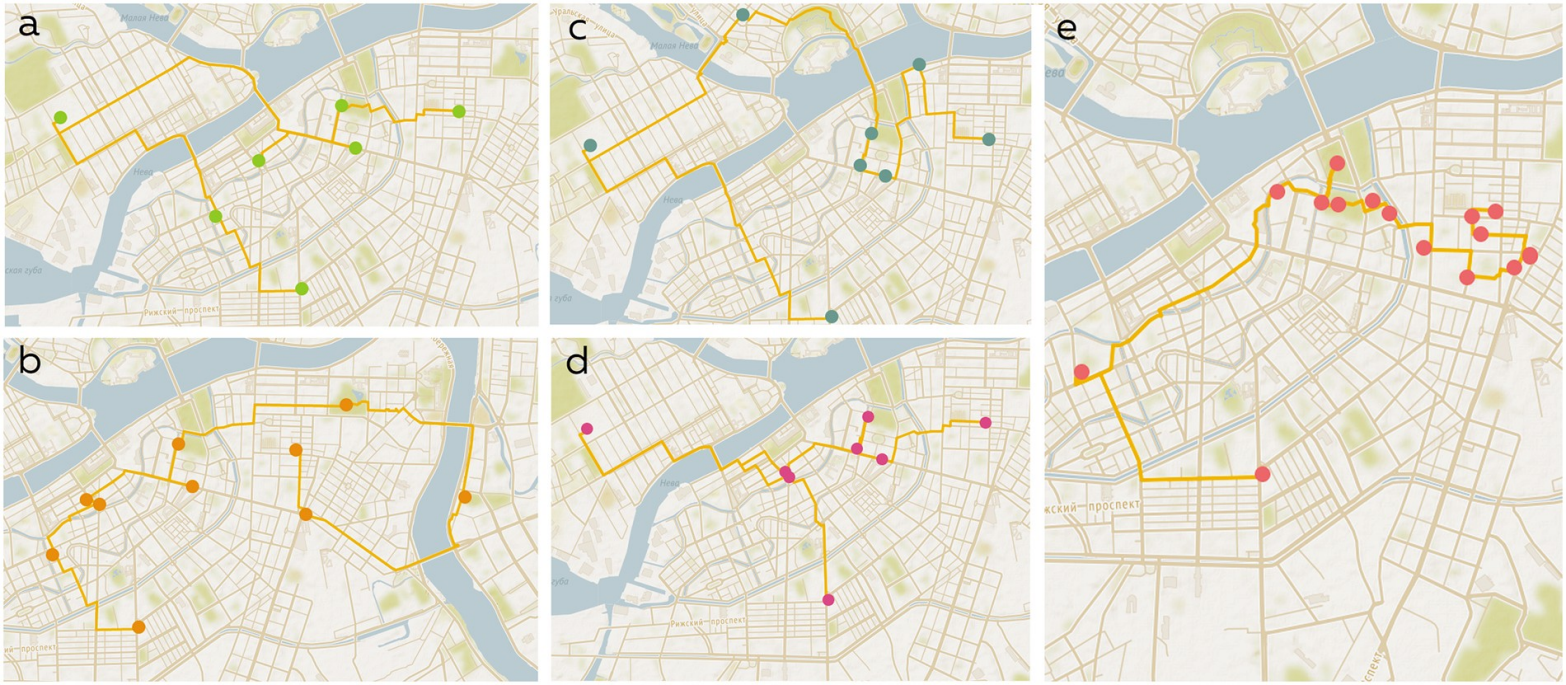
Comparison of automatic routes obtained by RGA. a—OP, b—OPCV, c—OPTW, d—TDOP, e—OPFP.

In this section we discuss results obtained from the dataset containing 5000 places. In Fig 4, routes obtained using RGA algorithm are presented. As can be seen from the maps, some locations are included in all first four itineraries. This happens due to the similar score function, which is used for all four problems. It can be noticed that solutions obtained for OP, OPCV, OPTW, and TDOP cover larger area than OPFP’s solution (Fig 4e). The high tightness of places in the solution of OPFP was ensured by the distance coefficient in score function. In contrast, other four routes consist of the most popular Instagram places despite their spatial scatter. Thus, it is clear that OPFP provides more convenient walking routes.

**Fig 5:**
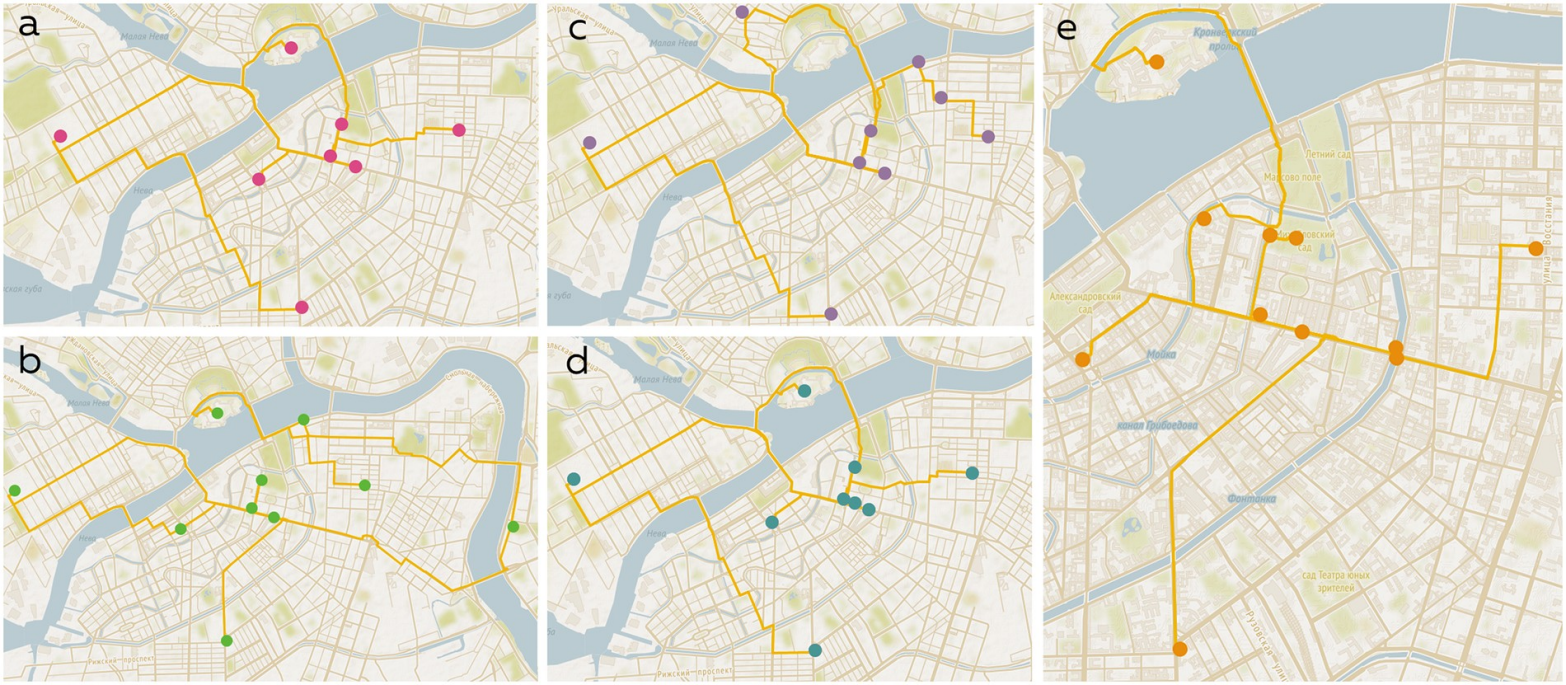
Comparison of automatic routes obtained by ACO. a—OP, b—OPCV, c—OPTW, d—TDOP, e—OPFP.

Fig 5 illustrates routes obtained using ACO algorithm. As it was mentioned in the previous section, ACO algorithm allows to get better results in terms of quality due to the higher variance of considered possible solutions. As can be seen from the maps, all resulting paths are shorter comparing to paths generated by RGA for corresponding problems. The result of solving OPFP is the most eventful itinerary among the others; this result is achieved due to the distance coefficient and popularity weights. More dense route will get a higher score than a route with one the most popular place using our dynamic score function. Thus, we can conclude that solving OPFP would allow not only to consider dynamic factors during the route construction but also to achieve more walkable resulting routes.

## Conclusion & future works

In this paper, we present an extension of classic OP—Orienteering Problem with Functional Profits (OPFP), where score of specific point depends on its characteristics, position in the route, and other points in the itinerary. For solving OPFP, we developed an open-source framework written in Go programming language (version 1.8.3) for solving orienteering problems, which utilizes four core components of OP (a set of points of interest, constraints, scoring function and solving algorithm) in its modular architecture. The modularity of our framework allows combining all components easily. It was demonstrated in our experiments through solving OP, OPCV, OPTW, TDOP along with OPFP. Experiments were conducted on a large multi-source dataset of Saint Petersburg, Russia, containing data from Instagram, TripAdvisor, Foursquare and official touristic website. Workstation was equipped by Intel(R) Core i7-3930K @ 3.20GHz processor, 32 GB RAM, Windows 7 SP1. Our framework is able to construct touristic paths for different OP types in less than one second for 100 points dataset, which outperforms known solutions.

Nevertheless, there are several directions for the further development of this work. One of possible direction for future research is studying different score functions. The one used in our examples served the goal to emphasize all popular places of different types. However, depending on researcher’s agenda score function can be modified; it can result in interesting discoveries about the city. With differentiation of PoIs’ set, score function and constraints, we are not bounded by domain-specific of OP family tasks. Thus, usage of FOPS can boost active development of OP application in other areas apart from tourism.
